# Androgen receptor gene expression in primary breast cancer

**DOI:** 10.1038/s41523-019-0142-6

**Published:** 2019-12-10

**Authors:** Neelima Vidula, Christina Yau, Denise Wolf, Hope S. Rugo

**Affiliations:** 1 Massachusetts General Hospital, 55 Fruit Street, Boston, MA 02114 USA; 2 University of California San Francisco, 1600 Divisadero Street, San Francisco, CA 94115 USA; 3 University of California San Francisco, 1600 Divisadero Street, San Francisco, CA 94115 USA

**Keywords:** Breast cancer, Tumour biomarkers

## Abstract

We studied androgen receptor (AR) gene expression in primary breast cancer (BC) to determine associations with clinical characteristics and outcomes in the I-SPY 1 study. AR was evaluated in I-SPY 1 (*n* = 149) using expression microarrays. Associations of AR with clinical and tumor features were determined using the Wilcoxon rank sum test (two-level factors) or the Kruskal–Wallis test (multi-level factors). We identified an optimal AR cut-point to maximize recurrence-free survival (RFS) differences between AR biomarker stratified groups, and assessed the association between the AR stratified groups and RFS using the Cox proportional hazard model. Pearson correlations between AR and selected genes were determined in I-SPY 1, METABRIC (*n* = 1992), and TCGA (*n* = 817). AR was lower in triple negative BC vs. hormone receptor positive (HR+)/HER2− and HER2+ disease (*p* < 0.00001), and lower in basal-like BC (*p* < 0.00001). AR was higher in grade I/II vs. III tumors (*p* < 0.00001), in patients >age 50 (*p* = 0.05), and in node negative disease (*p* = 0.006). Higher AR was associated with better RFS (*p* = 0.0007), which remained significant after receptor subtype adjustment (*p* = 0.01). AR correlated with expression of luminal, HER2, and steroid hormone genes. AR expression was related to clinicopathologic features, intrinsic subtype, and correlated with improved outcome.

## Introduction

Although breast cancer has conventionally been classified based on the presence or absence of the estrogen receptor (ER), progesterone receptor (PR), and human epidermal growth factor receptor 2 (HER2), recent studies have indicated that the genomic landscape of breast cancer extends far beyond these 3 receptors.^1^ Genomic profiling of tumor specimens has helped identify other targets that may serve as driver mutations for the development of breast cancer, contributing to the heterogeneity of this disease.^1–4^

The androgen receptor (AR) may be expressed in breast cancer. Lehmann and colleagues identified a subtype of triple-negative breast cancer (TNBC) characterized by the presence of the androgen receptor (AR) and expression of other luminal genes termed the luminal-androgen receptor (LAR) subtype. This expression-based subtype was subsequently confirmed by other authors.^1,2^

A better understanding of the relationship of AR, clinical characteristics, and patient outcomes is needed to further individualize patient care based on tumor biology. A recent study demonstrated that varying subtypes of TNBC may respond differently to neoadjuvant therapy.^5^ It is likely that varying subtypes within each of the conventional types of breast cancer (hormone receptor (HR) positive, HER2+, TNBC) may have different clinical and prognostic features, and AR expression may factor into this heterogeneity, across breast cancer subtypes. In this study, we evaluated associations between AR gene expression in primary breast cancer, clinical characteristics, and patient outcomes in a well characterized subset of patients with early stage breast cancer in the I-SPY 1 cohort. We also explored gene correlations with AR and selected other genes involved in the development of breast cancer, using three publically available databases including I-SPY 1 (Investigation of Serial Studies to Predict Your Therapeutic Response With Imaging and Molecular Analysis),^6,7^ METABRIC,^4^ and The Cancer Genome Atlas (TCGA).^8^

## Results

### I-SPY 1 tumor characteristics

Of the 149 patients with microarray data available, 25% (37) were TNBC, 30% (45) were HER2+, and 58% (86) were hormone receptor (HR)+. Of the patients with TNBC 86% were found to be basal by expression profiling.

### Association between AR expression and primary tumor characteristics

**Fig. 1:**
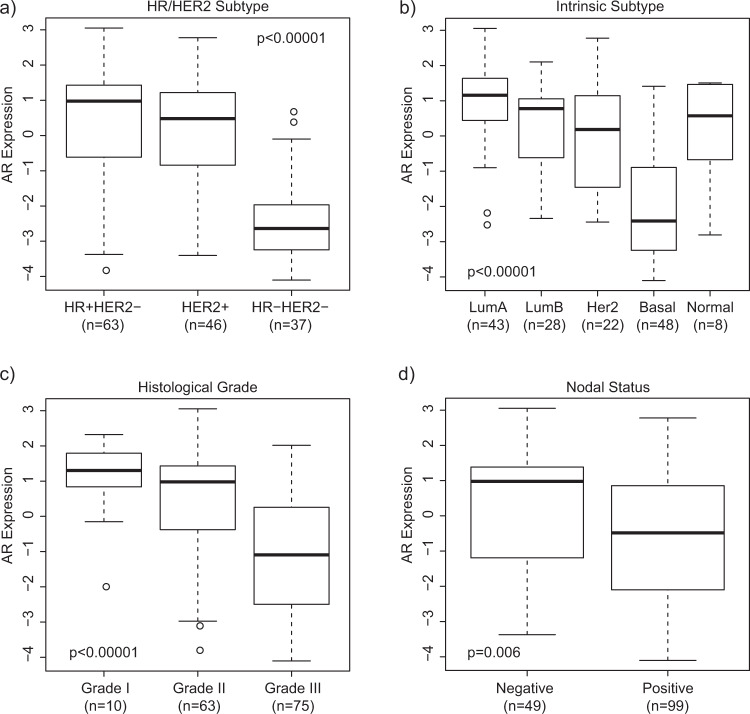
AR expression according to HR/HER2 subtype, intrinsic subtype, histological grade, and nodal status in I-SPY 1. Box plots show median-centered normalized AR gene expression levels stratified by **a** HR/HER2 status; **b** expression-based intrinsic subtype; **c** histological grade; and **d** nodal status. The line within boxes indicate the median AR expression; and boxes span the inter-quartile range (IQR). Whiskers span 1st quartile −1.5 × IQR and 3rd quartile +1.5 IQR; and outliers are represented by points.

In the I-SPY 1 dataset, AR expression was found to be significantly lower in TNBC (i.e. HR−/HER2−) than HR+/HER2− and HER2+ disease (Fig. 1a, *p* < 0.00001), as expected and concordant with previous literature.^9^ When evaluated by intrinsic subtype, AR expression was the lowest in basal breast cancer (Fig. 1b, *p* < 0.00001). Significantly higher AR expression was also observed in grade I/II versus grade III tumors (Fig. 1c, *p* < 0.00001), and was associated with older age >50 (median AR expression, age >50: 0.36 vs. age ≤50: −0.35, *p* = 0.05). These findings were also observed in METABRIC. Additionally, in I-SPY 1 higher AR expression was found in node negative versus node positive disease (Fig. 1d, *p* = 0.006); no association was seen with menopausal status (*p* = 0.70), clinical stage (*p* = 0.31), or lymphovascular invasion (*p* = 0.22).

### Association between AR expression with patient chemotherapy response and outcome

**Fig. 2:**
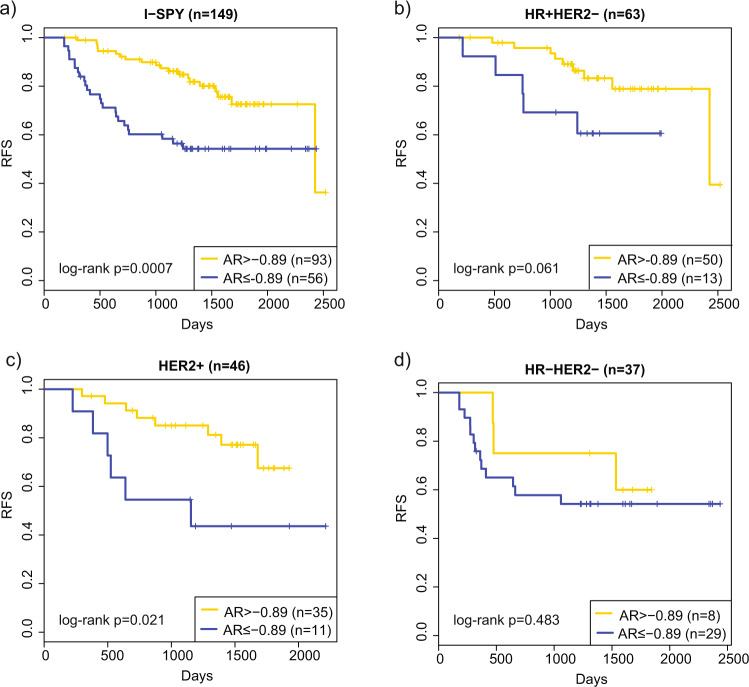
Association of AR expression with recurrence-free survival (RFS). I-SPY 1 patients were divided based on AR gene expression level into High vs. Low groups using an optimal cut point of −0.89. Kaplan–Meier survival plots of the AR High (gold) vs. Low (blue)groups within **a** all, **b** HR + HER2−, **c** HER2+, and **d** HR-HER2− patients are shown.

No significant association was observed between AR expression and pathologic complete response (pCR) following neoadjuvant chemotherapy (median AR expression, pCR: −0.81 vs. non-pCR: 0.24, *p* = 0.12). At an optimal cut off point that maximizes survival differences between AR-stratified groups (−0.89), higher AR expression (>−0.89) was associated with better recurrence-free survival (RFS) (Fig. 2a, log-rank *p* = 0.0007). In a multivariable Cox model adjusting for HR/HER2 subtypes, high AR expression remained significantly associated with better RFS (Wald test *p* = 0.01), suggesting that AR expression has prognostic value independent of receptor subtypes. The Kaplan–Meier survival plots of the AR-High vs. Low groups within each HR/HER2 subtypes are shown in Fig. 2b–d. High AR expression has a significant or strong trend for association with better outcomes within the HER2+ and HR+/HER2−, but not the HR-/HER2- subtype (likely due to the small number of HR-/HER2- patients with AR expression above −0.89).

### Gene correlations with AR expression

We determined correlations between the gene expression of AR and a set of 79 selected genes which may be involved in the development of breast cancer, using the I-SPY 1, METABRIC, and TCGA datasets in the population as a whole, and in the triple negative (TN) cohorts of METABRIC and TCGA. Table 1 shows the Pearson correlation coefficient of genes showing significant correlation with AR expression in ≥2 datasets.

**Table 1:** Gene correlations with AR in I-SPY 1, METABRIC, TCGA, and the triple negative (TN) cohorts of METABRIC (METABRIC TN) and TCGA (TGCA TN).

Category	Gene	I-SPY 1 (*n* = 149)	METABRIC (*n* = 1992)	METABRIC TN (*n* = 320)	TCGA (*n* = 817)	TCGA TN (*n* = 101)
*(A) Genes with positive correlations*						
Luminal	FOXA1	0.74	0.66	0.73	0.76	0.62
Luminal	GATA3	0.67	NA	NA	0.62	0.44
Luminal	AGR2	0.67	0.40	0.46	0.65	0.53
Luminal	PIP	0.59	NA	NA	0.44	0.53
Luminal	CA12	0.58	0.50	0.36	0.64	0.39
Luminal	ESR1	0.56	0.50	0.23	0.63	0.40
Luminal	PGR	0.50	0.31	NS	0.54	0.59
Luminal	TOX3	0.40	0.28	0.42	0.54	0.41
Luminal	MUC1	0.36	0.44	0.34	0.33	0.26
Luminal	DHRS2	0.31	0.25	0.61	0.35	0.49
Luminal	ITGB5	0.24	0.27	0.29	0.44	0.44
Luminal	CCND1	0.19	0.26	−0.07^NS^	0.35	0.11^NS^
Luminal	RET	0.20	0.25	0.33	0.42	0.38
HER	ERBB2	0.37	0.21	0.34	0.27	0.35
HER	ERBB3	0.31	0.50	0.34	0.51	0.16^NS^
HER	ERBB4	0.62	0.03^NS^	−0.06^NS^	0.63	0.50
Steroid Hormone	ADRA2A	0.49	0.20	0.27	0.43	0.50
Steroid Hormone	PTGER3	0.44	0.18	0.12^NS^	0.44	0.47
Steroid Hormone	GHR	0.41	0.28	0.54	0.46	0.42
Steroid Hormone	ADRB2	0.19	0.03^NS^	0.19	0.26	0.39
Other	TNFSF10	0.29	0.21	0.22	0.26	0.22^NS^
Other	KRAS	0.27	−0.03^NS^	−0.07^NS^	0.15	−0.05^NS^
Other	PTEN	0.08^NS^	0.14	0.05^NS^	0.42	0.33
*(B) Genes with negative correlations*						
Mesenchymal	CDK6	−0.53	−0.48	−0.29	−0.31	−0.12^NS^
Mesenchymal	EGFR	−0.35	−0.26	0.07^NS^	−0.22	0.01^NS^
Mesenchymal	PTGFR	−0.33	−0.18	−0.09^NS^	−0.24	−0.18^NS^
Basal	FOXC1	−0.51	−0.50	−0.58	−0.66	−0.61
Basal	MYC	−0.46	−0.31	−0.41	−0.29	−0.24
Basal	HORMAD1	−0.42	−0.42	−0.42	−0.63	−0.37
Basal	SERPINB5	−0.40	−0.38	−0.40	−0.40	−0.41
Basal	SOX10	−0.38	−0.46	−0.51	−0.36	−0.36
Basal	ELF5	−0.29	−0.38	−0.37	−0.44	−0.36
Basal	VTCN1	0.15^NS^	−0.10	−0.32	−0.10	−0.23
Basal	SOX6	N/A	−0.15	−0.19	−0.29	−0.25
Basal	SOX8	−0.36	−0.36	−0.33	−0.53	−0.46
Basal	TUBB2B	−0.12^NS^	−0.27	−0.28	−0.21	−0.08^NS^
Basal	KIT	−0.11^NS^	−0.28	−0.28	−0.12	−0.20^NS^
Basal	KRT5	−0.24	−0.37	−0.27	−0.29	−0.21^NS^
Basal	KRT14	−0.18	−0.23	−0.25	−0.18	−0.16^NS^
Immune	FOXP3	−0.29	0.00^NS^	−0.07^NS^	−0.18	−0.01^NS^
Immune	RARRES1	−0.15^NS^	−0.48	−0.23	−0.43	−0.17^NS^
Immune	CXCL9	0.11^NS^	−0.24	−0.02^NS^	−0.18	−0.06^NS^
Immune	CXCL10	−0.05^NS^	−0.32	−0.18	−0.32	−0.22^NS^
Immune	STAT5A	−0.16^NS^	−0.15	−0.07^NS^	−0.077	−0.07^NS^
Immune	STAT1	−0.05^NS^	−0.21	−0.13	−0.09	−0.00^NS^
Immune	CTLA4	−0.11^NS^	−0.32	−0.12^NS^	−0.28	−0.06^NS^
Immune	PSMB9	−0.17^NS^	−0.29	−0.16	−0.34	−0.21^NS^
Immune	LCK	−0.17^NS^	−0.24	−0.06^NS^	−0.24	−0.09^NS^
Immune	CD2	0.14^NS^	−0.23	−0.02^NS^	−0.18	0.02^NS^
Immune	PDCD1	−0.04^NS^	−0.20	−0.03^NS^	−0.28	−0.08^NS^
DNA repair	TOP2A	−0.12^NS^	−0.19	−0.34	−0.17	−0.25
DNA repair	PARPi−7	NA	−0.21	−0.16	−0.42	−0.22
Other	CRYAB	−0.18	−0.40	−0.32	−0.45	−0.30

Significant correlations (*p* < 0.05) with AR expression seen in ≥2 datasets are depicted here with the correlation coefficient (*r*)

*NS* non-significant, *NA* not available in dataset

AR gene expression positively correlated with the expression of multiple luminal genes including FOXA1, GATA3, AGR2, PIP, CA12, ESR1, PGR, TOX3, MUC1, DHRS2, ITGB5, CCND1, and RET as well as with the expression of steroid hormone genes such as ADRA2A, PTGER3, GHR, and ADRB2. In addition, AR gene expression positively correlated with the expression of HER2 pathway genes including ERBB2, ERBB3, and ERBB4, and the expression of TNFSF10, KRAS, and PTEN. Most these gene correlations were also apparent in the TN cohorts.

Significant inverse gene correlations with AR were noted with the basal genes FOXC1, MYC, HORMAD1, SERPINB5, SOX10, ELF5, VTCN1, SOX6, SOX8, TUBB2B, KIT, KRT5, and KRT14; in most cases, these were observed in the TN cohorts as well. AR gene expression also inversely correlated with the expression of DNA damage repair genes, TOP2A and the PARPi-7 gene signature in the population as a whole, and in the TN cohorts alone. Finally, a significant inverse correlation between AR gene expression and the expression of CRYAB was observed, both in the population as a whole, and in the TN cohorts alone.

In addition, significant inverse correlations of AR expression with mesenchymal genes, CDK6, EGFR, and PTGFR, and immune genes FOXP3, RARRES1, CXCL9, CXCL10, STAT5A, STAT1, CTLA4, PSMB9, LCK, CD2, and PDCD1 were noted; however, these correlations were generally not seen in the TN cohorts, suggesting that this may be related to HR/HER2 status rather than AR expression.

## Discussion

The androgen receptor may characterize a discrete subtype of breast cancer. In this study, we analyzed gene expression in the AR pathway in patients enrolled in I-SPY 1, and utilized the METABRIC and TCGA datasets for validation. In both the I-SPY 1 and METABRIC datasets, we noted a lower expression of AR in TNBC than in HR+/HER2− and HER2+ disease, and the lowest expression in basal type breast cancer. Similarly, other authors have noted increased expression of AR in HR+ than ER- disease,^10–13^ and a lower expression of AR in TNBC.^9^

We also observed that AR expression correlated with features of less aggressive disease when evaluated across subtypes, with higher expression in grade I/II versus III tumors in both I-SPY 1 and METABRIC, and higher expression in node negative versus node positive tumors in I-SPY 1. Our findings are concordant with those of other authors who have demonstrated increased AR expression in well differentiated tumors^11,14^ and node negative disease.^13,15^ Additionally, higher AR expression correlated with older age (>50) in both I-SPY 1 and METABRIC, concordant with other datasets.^14,16^ We did not find a correlation of increased AR expression with lower disease stage in I-SPY 1, although others have observed this finding.^15^

In concordance with the association of AR expression with lower risk disease, we demonstrated that higher AR expression correlated with better RFS in the I-SPY 1 dataset. The prognostic significance is particularly interesting as the patients in the I-SPY 1 cohort were treated with neoadjuvant chemotherapy, and AR expression was not associated with higher rates of pathologic complete response, suggesting that the prognostic impact may not be as tightly related to chemotherapy response. The association between AR and receptor subtypes may be a potential confounding factor as the subtypes have distinct pathologic complete response rates and prognosis. Indeed, AR expression was high in HR+/HER2− patients who had the lowest pathologic complete response rates but the best prognosis.^6^ Nevertheless, AR remained an independent prognostic factor in a Cox model adjusting for receptor subtypes.

However, a caveat to these findings is that given the sample size of the I-SPY1 dataset (*n* = 149), the number of patients with various subtypes is small, limiting our ability to draw firm conclusions about the impact of AR gene expression on outcomes. Additionally, the cut point used for AR gene expression in this analysis was data-derived and therefore the outcome data are hypothesis generating and require validation in a different cohort of patients. We recognize that I-SPY1 has a higher proportion of high risk patients (due to the eligibility criteria of ≥3 cm and the subtype distribution has a lower proportion of HR+/HER2− tumors than one would expect based on the general population. However, the association between AR gene expression and subtypes (receptor or intrinsic), grade, and age were observed in both the I-SPY 1 and METABRIC, which has a much higher proportion (71%) of HR+/HER2− cases. Based on this assessment, it is unlikely these findings are attributable to the skewed subtype distribution of the I-SPY 1 cases.

Nevertheless, the majority of literature also suggests that AR positivity may be associated with better outcomes. Several recent studies have demonstrated an improvement in both disease free survival (DFS) and/or overall survival (OS) in AR positive tumors.^12,14,17–21^ However, some authors have demonstrated the opposite finding, i.e. that AR over-expression is associated with worse survival outcomes, including an association with poor OS in one study^22^ (149 patient study), and worse DFS in AR + TNBC in 2 studies (Asano et al.^9^ (61 patient study) and Jiang et al.^23^ (137 patient study)), with all of these studies utilizing immunohistochemical (IHC) testing for AR assessment. In addition to population differences and variations in treatment patterns, a possible explanation for these discordant findings is variability in the IHC methodology used to assess AR expression. The majority of the studies in the literature have relied upon IHC to assess tumor AR status. Kim’s meta-analysis^17^ exemplifies the variability that may occur in assessing AR positivity using IHC expression, as the 11 studies utilizing IHC that were included defined expression in several different ways. Clearly there is not a consensus definition for AR positivity by IHC, and in prospective clinical trials, the optimal approach to defining AR positivity is also still being debated, with recent clinical trials defining AR+ disease as ≥0–10% by IHC or by utilizing a genomic profiling assay.^24–26^ An advantage of the I-SPY 1 database is that AR positivity was defined using expression microarrays, which have less variability than non-standardized IHC analyses.

Consistent with our findings that AR expression is lower in triple negative and basal subtype breast cancer, using the I-SPY 1, METABRIC, and TCGA datasets (defining a positive gene correlation as one seen in ≥2 datasets), we were able to demonstrate that the gene expression of AR positively correlates with the expression of a multitude of luminal genes. As expected, AR expression also correlated with the gene expression of other steroid hormones. AR also anti-correlated with basal, mesenchymal, and immune genes. Many of these observations were also noted in the TNBC cohorts of METABRIC and TCGA, suggesting that AR receptor positivity may define a subset of TNBC with luminal features consistent with the previously described LAR subtype of TNBC.^1,10^

Understanding AR+ disease is particularly important in TNBC where the mainstay of treatment is chemotherapy, and few targeted therapies are available. A recent phase II study evaluated the use of bicalutamide, an androgen antagonist, in patients with AR+/ER- metastatic breast cancer, defining AR positivity as IHC >10%.^24^ Altogether 424 patients with ER-/PR- breast cancer were screened of which 12% were AR positive. A clinical benefit rate (CBR) of 19% (95% CI: 7–39%) was noted in the 26 evaluable patients, and the median PFS was 12 weeks (95% CI: 11–22 weeks). A second phase II study evaluated enzalutamide, an androgen receptor inhibitor, in advanced AR + TNBC.^26^ In the 118 intent-to-treat population (AR >0% by IHC), the CBR at 16 weeks was 25% (95% CI: 17–33%) and median PFS was 2.9 months (95% CI: 1.9–3.7 months), and these endpoints were slightly improved in the evaluable population of 78 patients (AR ≥10% by IHC) where the CBR at 16 weeks was 33% (95% CI: 23–45%) and the median PFS was 3.3 months (95% CI: 1.9–4.1 months). Using a genomic assay for AR expression (PREDICT AR+), 56 patients with a positive PREDICT AR assay had a clinical benefit rate of 39% at 16 weeks, and a median PFS of 16.1 weeks.^25^ A third study evaluated seviteronel, a selective CYP17 lyase and AR inhibitor in advanced breast cancer, including TNBC, demonstrating the initial safety and efficacy in TNBC (2 patients with clinical benefit at 4 months).^27^ These findings suggest potential value for therapies targeted to AR. While response rates may be low, some of them are durable which is encouraging for TNBC. Additional studies are exploring orteronel in TNBC disease,^28^ as well as combination therapy in AR+ TNBC, including a study of enzalutamide and paclitaxel in the pre-operative setting^29^ and one study evaluating enobosarm, a selective androgen receptor modulator, with pembrolizumab, an immunotherapy agent.^30^

However, better definitions of AR positivity are clearly required to identify patients likely to benefit from AR targeted therapies. Our work establishes the feasibility in utilizing gene expression microarrays for evaluating AR expression in tumor tissue, and this is one potential platform that could be considered in future trials, as it may circumvent the issue of non-standardization of immunohistochemical assays to identify patients who may benefit from AR blockade. However, further research is needed to compare AR IHC and genomic assays. Unfortunately, this could not be done using the I-SPY1 dataset as AR IHC results are not available for this cohort. However, we noted a 0.73 correlation coefficient between AR protein level as assessed using reverse phase protein array (RPPA) and AR gene expression levels in TCGA cohort. Additional research is needed to help AR gene expression levels translate into routine clinical practice, similar to other expression-based multi-gene signatures such as the Oncotype and MammaPrint which are used in clinical decision making. The development of a validated AR gene expression signature or validated AR IHC threshold may enable clinicians to identify those patients with metastatic breast cancer who may benefit from AR targeted therapies, and for earlier stage disease, to aid in risk prognostication.

## Methods

Due to the retrospective nature of this study using only publicly available data, ethics approval for the study was not required.

### Study population

The I-SPY 1 (Investigation of Serial Studies to Predict Your Therapeutic Response With Imaging and Molecular Analysis) study was a multicenter initiative performed by the American College of Radiology Imaging Network (ACRIN), Specialized Programs of Research Excellence (SPORE), and the Cancer and Leukemia Group B (CALGB). The design of this trial is described elsewhere.^6,7^ In summary, I-SPY 1 enrolled patients with early stage breast cancer and ≥3.0 cm of disease in breast, without evidence of distant metastases. Patients underwent a core biopsy of their breast tumor at the time of enrollment, then during, and after completing neoadjuvant chemotherapy. Patients received 4 cycles of neoadjuvant anthracycline plus cyclophosphamide chemotherapy with 95% receiving a taxane prior to undergoing surgery. Starting in 2005, patients with HER2+ disease also received trastuzumab. Following surgery, patients received adjuvant chemotherapy, trastuzumab, hormonal therapy (given to all patients with HR+ disease), and/or radiation per physician discretion.

### I-SPY 1 gene expression dataset

For 149 patients enrolled in I-SPY 1 (GSE22226), high quality gene expression data, assayed with the Agilent 44K arrays, was generated from the pre-neoadjuvant treatment biopsies; the clinical features of these 149 patients did not vary significantly from the total sample size of 221 patients evaluable. Previous studies have described the methodology by which the microarray data was generated and processed, and the manner in which molecular profiling was performed.^6^ Specifically, the expression of AR was taken as the median-centered log2-scaled loess-normalized expression value of the A_23_P113111 probe.

### METABRIC gene expression dataset

Normalized expression matrices of the METABRIC discovery and validation cohorts, generated on the HT-12 v3 platform were obtained from http://www.ebi.ac.uk/ega/ (accession number EGAS00000000083).^4^ ComBat was used for batch adjustment when combining the discovery and validation cohorts to yield a dataset containing 1992 samples. Data was annotated using the corresponding array annotation file from GEO, and genes represented by multiple probes are collapsed by averaging. The normalized, batch-adjusted, gene-level expression data was median-centered prior to analysis.

### TCGA gene expression dataset

Normalized gene-level expression data, assayed by RNA-sequencing, for 817 primary breast cancers analyzed as part of the TCGA program was obtained from the TCGA data portal website (http://tcga—data.nci.nih.gov/tcga). Details of the data processing can be found in Ciriello et al.^8^

### Association between AR primary tumor expression, clinical and tumor characteristics, chemotherapy response, and outcome

Associations between AR expression and clinical and tumor characteristics were assessed using the Wilcoxon rank sum test (for two-level factors) or the Kruskal-Wallis test (for multi-level factors). The clinical characteristics assessed in both I-SPY and METABRIC datasets included: patient age (≤50 or >50), menopausal status, clinical stage (stage I/I vs. III or inflammatory), tumor HR and HER2 status, intrinsic subtype (basal vs. non basal), nodal status (node positive vs. node negative), histologic grade (grade I/II vs. grade III).

In addition, in the I-SPY 1 dataset, the associations between AR and tumor lymphovascular invasion (presence vs. absence), as well as chemotherapy response (pathologic complete response vs. not) were assessed. We also evaluated the association of AR expression and recurrence-free survival (RFS). First, we identified an optimal AR cut-point which maximizes the survival difference between the AR-stratified groups. Kaplan Meier survival curves were constructed to visualize the survival differences between AR-stratified groups in the overall I-SPY cohort and within HR/HER2 subtypes. Significance in curve separation was assessed using a log-rank test. A multivariate Cox analysis was used to evaluate the association between AR and RFS, adjusting for HR/HER2 subtype.

### Determination of external gene correlations with AR expression

We determined correlations between the gene expression of AR and a selected set of genes that may be involved in the development of breast cancer, including genes shown to be associated with the luminal, mesenchymal, basal, and immune subtypes of TNBC,^2^ as well as DNA damage repair deficiency genes including TDG and a seven gene signature shown to be associated with response to the poly ADP ribose polymerase (PARP) inhibitor olaparib, the PARPi-7.^31^ This signature was calculated on median-centered expression data in simplified form as PARPi-7 = −0.5320*BRCA1 + 0.5806*CHEK2 + 0.0713*MAPKAPK2 − 0.1396*MRE11A − 0.1976*NBN − 0.3937*TDG −0.2335*XPA. Gene correlations were determined in all 3 datasets, I-SPY 1, METABRIC, and TCGA, as well as in the triple negative (TN) subsets of the 2 larger datasets, METABRIC and TCGA. Gene associations were determined using Pearson correlations with the Benjamini Hochberg correction for multiple testing (*p* < 0.05).

### Reporting summary

Further information on research design is available in the Nature Research Reporting Summary linked to this article.

## Supplementary information

Reporting Summary

## Data Availability

The I-SPY 1, METABRIC, and TCGA datasets are publicly available as described in “Methods” above. I-SPY 1 datasets were accessed from the NCBI Gene Expression Omnibus (GEO) repository: https://identifiers.org/geo:GSE22226. TCGA gene expression data were accessed from the cBioPortal for CancerGenomics: https://identifiers.org/cbioportal:brca_tcga_pub2015. METABRIC gene expression data were accessed from the European Genome-phenome Archive (EGA): https://identifiers.org/ega.dataset:EGAD00010000210 and https://identifiers.org/ega.dataset:EGAD00010000211. The data generated and analyzed during this study are described in the following metadata record: 10.6084/m9.figshare.10275182.^32^

## Code availability

Analysis is performed using R v3.4.2;^33^ and code is available upon request.
